# Do 3D Face Images Capture Cues of Strength, Weight, and Height Better than 2D Face Images do?

**DOI:** 10.1007/s40750-021-00170-8

**Published:** 2021-08-26

**Authors:** Iris J Holzleitner, Alex L Jones, Kieran J O’Shea, Rachel Cassar, Vanessa Fasolt, Victor Shiramizu, Benedict C Jones, Lisa M DeBruine

**Affiliations:** 1 Institute of Neuroscience & Psychology, University of Glasgow, Glasgow, Scotland; 2 Department of Health and Social Sciences, University of the West of England, Bristol, England; 3 Department of Psychology, Swansea University, Swansea, Wales; 4 School of Psychological and Health Sciences, University of Strathclyde, Glasgow, Scotland

**Keywords:** Face perception, Attributions, Accuracy, Formidability

## Abstract

**Objectives:**

A large literature exists investigating the extent to which physical characteristics (e.g., strength, weight, and height) can be accurately assessed from face images. While most of these studies have employed two-dimensional (2D) face images as stimuli, some recent studies have used three-dimensional (3D) face images because they may contain cues not visible in 2D face images. As equipment required for 3D face images is considerably more expensive than that required for 2D face images, we here investigated how perceptual ratings of physical characteristics from 2D and 3D face images compare.

**Methods:**

We tested whether 3D face images capture cues of strength, weight, and height better than 2D face images do by directly comparing the accuracy of strength, weight, and height ratings of 182 2D and 3D face images taken simultaneously. Strength, height and weight were rated by 66, 59 and 52 raters respectively, who viewed both 2D and 3D images.

**Results:**

In line with previous studies, we found that weight and height can be judged somewhat accurately from faces; contrary to previous research, we found that people were relatively inaccurate at assessing strength. We found no evidence that physical characteristics could be judged more accurately from 3D than 2D images.

**Conclusion:**

Our results suggest physical characteristics are perceived with similar accuracy from 2D and 3D face images. They also suggest that the substantial costs associated with collecting 3D face scans may not be justified for research on the accuracy of facial judgments of physical characteristics.

## Introduction

There now exists a large literature examining the extent to which physical characteristics, such as formidability and health, can be accurately assessed from face images (for reviews of this literature see De Jager et al., 2018 and Puts, 2010). The results of such studies have implications for evolutionary theories of the signal value of facial characteristics in both human mate choice and intrasexual competition (De Jager et al., 2018; Puts, 2010).

Studies on this topic typically employ two-dimensional (2D) face images as stimuli (Coetzee et al., 2009, 2010; Re et al., 2013; Tinlin et al., 2013; Sell et al., 2008). However, some studies have used three-dimensional (3D) face images because they may contain cues that are not captured well in 2D face images and, therefore, are likely to have greater ecological validity (Holzleitner et al., 2014; Re et al., 2012). The equipment needed to obtain high quality 3D face images is considerably more expensive than the equipment needed to obtain high quality 2D face images. However, it is not known whether this additional cost is warranted (i.e., it is not known whether 3D face images capture cues of physical characteristics better than 2D face images do). The current study investigates this issue by comparing the accuracy of perceptions of strength, weight, and height from 2D and 3D face images.

Several studies have reported positive correlations between measures of upper-body strength, such as handgrip strength, and perceptual judgments (i.e., ratings) of strength from both 2D face photographs (Sell et al., 2008) and 3D face images (Holzleitner & Perrett, 2016). Studies have also reported positive correlations between body mass index (BMI) and ratings of weight from 2D face photographs (Coetzee et al., 2009, 2010; Tinlin et al., 2013) and 3D face images (Holzleitner et al., 2014). Other studies have reported positive correlations between height and ratings of height from 2D face photographs (Re et al., 2013) and 3D face images (Re et al., 2012; but see also Holzleitner et al., 2014). These studies suggest strength, weight, and height can be judged somewhat accurately from face images, but no studies have yet directly compared the effect of stimulus type (2D versus 3D face images) on the accuracy of perceptions of these (or any other) traits. While some previous work has used both 2D and 3D images, these studies have focused on testing attractiveness and formidability ratings only (Tigue et al., 2012; Trebicky et al., 2018), or similarities in face recognition (e.g., Eng et al., 2017) and morphological measurements (e.g., Hill et al., 2017).

In light of the above, we compared the effect of stimulus type (2D versus 3D face images) on the accuracy of perceptions of strength, weight, and height from face images. We tested three specific hypotheses.

### Hypothesis 1

Handgrip strength would be positively and significantly correlated with strength ratings of face images and this correlation would be significantly stronger for ratings of 3D than 2D images.

### Hypothesis 2

Height would be positively and significantly correlated with height ratings of face images and this correlation would be significantly stronger for ratings of 3D than 2D images.

### Hypothesis 3

BMI would be positively and significantly correlated with weight ratings of face images and this correlation would be significantly stronger for ratings of 3D than 2D images.

## Methods

### Face Stimuli

Stimuli were 2D and 3D face images of 119 women and 63 men (mean age = 24.0 years, SD = 8.4 years)^1^. These men and women first cleaned their face with hypoallergenic face wipes to remove any make-up. Face photographs were taken a minimum of 15 minutes later against a constant background and under standardized diffuse lighting conditions. The men and women were instructed to pose with a neutral expression. Camera-to-head distance (90 cm) and camera settings were held constant. Six photographs of each individual were taken simultaneously from different angles using a DI3D system (www.di4d.com) with six standard digital cameras (Canon EOS100D with Canon EF 50 mm f/1.8 STM lenses). These images have been collected as part of an ongoing project on 2D and 3D kinship cues. More information on the image collection procedure, including an example of the collected image data as well as a schematic drawing of the set up can be found at https://osf.io/gs5wm/.

The front-view face images as captured by the top middle camera were used as the 2D images. 3D images were generated using DI3Dview (version 6.8.9), which creates both a texture map in the BMP file format (exported at a resolution of 1MP) as well as a 3D mesh from the raw data that was exported in the Wavefront OBJ file format. Both 2D and 3D images were Procrustes-aligned prior to rating based on 132 landmarks and 55 landmarks respectively, to remove differences in alignment and size.

To make 2D and 3D images as comparable as possible, 3D face images were rendered with a perspective/field of view equivalent to that of 2D images. Details on our method to ensure fields of view between 2D and 3D images would be as close to each other as possible can be found in the supplemental material. 3D face images were rendered to rotate laterally from −45 to +45° in steps of 2.25° and were displayed at 18 frames per second as M4V movies^2^. To ensure viewing times of 2D and 3D stimuli were equivalent, 2D images were also compiled as (static) M4V movies with the same number of frames as 3D stimuli.

**Fig. 1:**
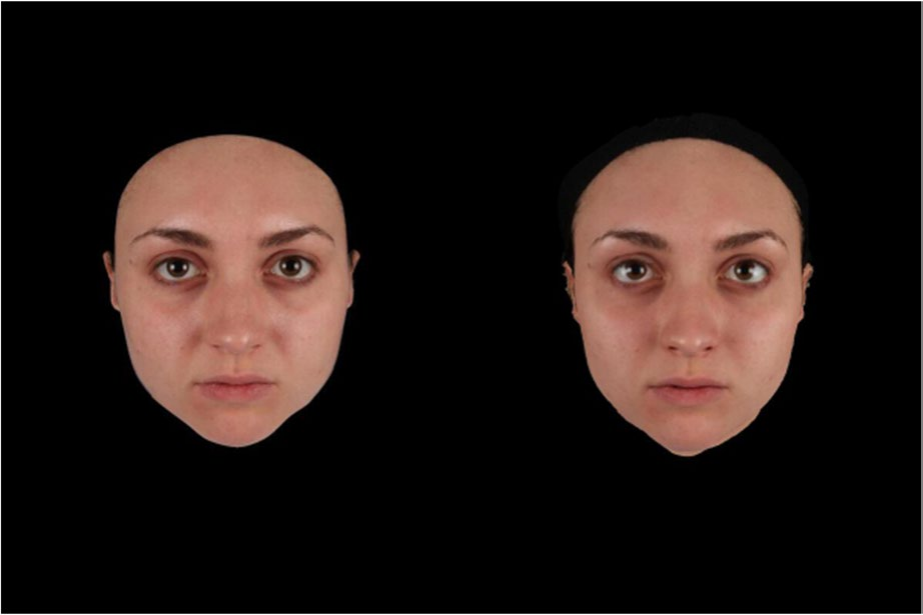
Example of one of the stimulus faces in 2D (left) and “3D” (right)

Both 2D and 3D face images were masked so that hairstyle and clothing were not visible and presented against a black background at a size of 600 x 800 pixel. See Figure 1 for an example frame from the 2D and 3D movies of one of our stimulus faces.

### Body Measures

Height and weight were measured from each participant and used to calculate BMI for each participant. Height was measured using a wall-mounted stadiometer. Weight was measured using a medical-grade seca 761 flat scale. Participants’ handgrip strength was measured from their dominant hand three times using a T. K. K. 5001 Grip A dynamometer. Following Fink et al. (2007) and Han et al. (2018), the highest recording from each participant was used in analyses. These body measures were taken when the images were obtained.

### Ratings

Participants were randomly allocated to rate the faces for strength, weight, or height using 1 (*not very strong/heavy/tall*) to 7 (*very strong/heavy/tall*) scales. Each participant rated the images for one trait only but rated both the 2D and 3D images for that trait.

We had originally planned to present each rater with all stimulus faces in a lab setting. Due to COVID-19, we had to move the rating study online. To alleviate experimental attrition, we split the stimulus set into three random subsets of 60 and 61 stimulus faces respectively (thereby reducing the number of ratings for each participant from 364 to 120 or 122 ratings). Each subset contained the same ratio of female and male faces. 2D and 3D faces were presented in separate blocks of trials; due to a mistake by the first author, male and female faces were not presented in separate blocks as originally planned. Trial order in each block was fully randomized and block order was also fully randomized.

Raters were recruited from the University of Glasgow’s Psychology Participant Panel. We had originally aimed to recruit twenty raters to rate each trait (i.e., 60 raters in total, approximately half men and half women, all aged between 16 and 40 years of age). Simulations suggested that this number of raters would be sufficient to produce high Cronbach’s alphas and stable averages for ratings of most traits (DeBruine & Jones, 2018). Having split the stimulus set into three subsets, we continued to aim for 20 raters for each trait, but now also for each face, i.e. 180 raters in total.

### Data Quality Checks

One hundred and ninety-three participants completed the study. Ratings from participants who indicated that they recognized any of the faces were removed from the dataset prior to any analyses. Twenty-two participants were removed from the dataset for this reason. We had also planned to remove ratings from participants who gave the same score for more than 75% of trials prior to any analyses; no participants had to be removed for this criterion. Thus, data from 171 participants, some of which completed the study for more than one trait, was analyzed.

We had planned to analyze data for a given trait only if the Cronbach’s alphas for ratings of 3D and 2D images were both greater than 0.8. Table 1 shows the final sample size for each trait and subset, as well as standardized Cronbach’s alpha and 95% CIs.

**Table 1:** Number of raters and standardized Cronbach’s alpha [95% CI] for each trait and stimulus subset

Trait	Subset	N	Cronbach’s alpha (2D)	Cronbach’s alpha (3D)
Strength	1	24	0.89 [0.84, 0.93]	0.90 [0.85, 0.93]
	2	21	0.88 [0.83, 0.92]	0.86 [0.78, 0.90]
	3	21	0.84 [0.76, 0.89]	0.84 [0.77, 0.89]
Height	1	20	0.92 [0.89, 0.95]	0.93 [0.89, 0.95]
	2	20	0.93 [0.90, 0.95]	0.94 [0.91, 0.96]
	3	16	0.93 [0.89, 0.95]	0.92 [0.89, 0.95]
Weight	1	16	0.94 [0.91, 0.96]	0.92 [0.88, 0.94]
	2	17	0.93 [0.90, 0.95]	0.96 [0.94, 0.97]
	3	19	0.94 [0.91, 0.96]	0.95 [0.92, 0.96]

### Analyses

Handgrip strength, height, and BMI were analyzed in separate multilevel models (see https://osf.io/wz5nc/ for full analysis code). In each analysis, the relevant rating (strength, height, or weight), averaged across all raters, was the outcome variable. Each rating was linearly rescaled from a 1–7 range to a −0.5 to 0.5 range in order to achieve convergence of the mixed effects models, but the estimates reported below have all been converted back to the 1–7 scale. The corresponding body measurement (handgrip strength, height, and BMI), centered on their means and scaled to range between −5. and +.5, were included as a predictor, along with sex of face (male or female, effect-coded) and stimulus type (2D or 3D image, effect-coded). The interaction between stimulus type and the relevant body measurement was also included in the model. The model also included a by-stimulus random intercept and by-stimulus random slope for stimulus type. For each hypothesis, average ratings more than three standard deviations above or below the mean for the relevant sex were excluded from the regression analysis. As the focus of this study lay in investigating accuracy of judgments across 2D and 3D stimuli and we saw no strong rationale to predict sex differences in accuracy of the investigated judgments, we did not analyze effects of rater sex.

## Results

We hypothesized that for each of the three traits under investigation (handgrip strength, height, BMI), anthropometric measurements would be positively and significantly correlated with perceptual ratings (as indicated by significant positive main effects of anthropometric measures), and that this correlation would be higher for 3D than 2D images (as indicated by a significant positive interaction between anthropometric measure and stimulus type).

### Model 1: Strength

**Fig. 2:**
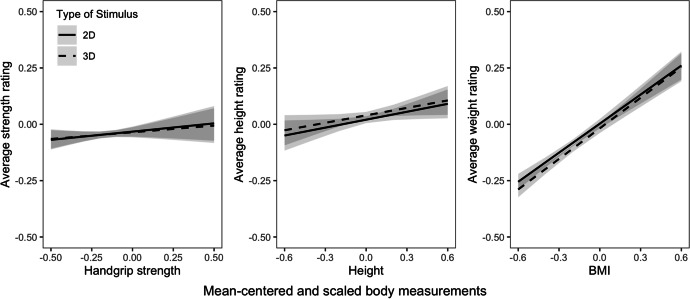
The interaction effect of stimulus type (2D or 3D) and measured traits (handgrip strength, height and BMI) on the respective perceptual ratings. As opposed to our predictions, accuracy of ratings did not differ between 2D and 3D stimuli for either of the tested traits

Full results for Model 1 are shown in Table 2. The mean strength rating was 3.90. There was a significant positive effect of stimulus gender (estimate = 0.11, 95% CI = [0.07, 0.16], *p* < .001), indicating that male stimulus faces were rated 0.68 points higher on strength on the 7-point scale than female stimulus faces. No other effects were significant (all *|t|≤*1.14, all *p* ≥ .255). These results provide no evidence for our hypothesis that strength can be judged from faces and can be judged more accurately from 3D images than 2D images (see also Figure 2).

**Table 2:** Results from hypothesis testing. Predicting perceptual ratings with respective anthropometric measurements (“trait value”), stimulus gender and stimulus type (2D vs 3D)

Trait	Term	Estimate	2.5%	97.5%	df	*t*	*p*
Strength	Intercept	−0.02	−0.04	0.00	179	−1.75	.082
	Stimulus gender	0.11	0.07	0.16	179	5.23	<.001
	Trait value	0.07	−0.05	0.18	179	1.14	.255
	Stimulus type	0.00	−0.01	0.01	180	−0.56	.577
	Trait value x stimulus type	−0.01	−0.06	0.03	180	−0.61	.539
Height	Intercept	0.06	0.04	0.07	177	6.80	<.001
	Stimulus gender	0.18	0.13	0.22	177	7.87	<.001
	Trait value	0.11	0.01	0.22	177	2.15	.033
	Stimulus type	0.02	0.01	0.03	178	4.54	<.001
	Trait value x stimulus type	−0.01	−0.05	0.03	178	−0.33	.744
BMI	Intercept	0.00	−0.02	0.02	179	−0.16	.875
	Stimulus gender	0.04	0.01	0.07	179	2.56	.011
	Trait value	0.44	0.37	0.51	179	12.01	<.001
	Stimulus type	−0.02	−0.03	−0.01	180	−3.06	.003
	Trait value x stimulus type	0.02	−0.03	0.07	180	0.88	.381

### Model 2: Height

Full results for Model 2 are shown in Table 2. The mean height rating was 4.35. There was a significant positive effect of stimulus gender (estimate = 0.18, 95% CI = [0.13, 0.22], *p* < .001), indicating that male stimulus faces were rated 1.06 points higher on height than female stimulus faces. There was also a significant positive effect of actual height (estimate = 0.11, 95% CI = [0.01, 0.22], *p* = .033), indicating that height was perceived with some accuracy: a difference of body height equivalent to the sample range led to an increase in height ratings by 0.68 points on the 7-point scale. The model also showed a significant positive effect of stimulus type (estimate = 0.02, 95% CI = [0.01, 0.03], *p* < .001), indicating that 3D faces received slightly higher height ratings than 2D faces (0.12 points). The interaction of actual height and stimulus type was not significant (estimate = −0.01, 95% CI = [−0.05, 0.03], *p* = .744). These results provide evidence for our hypothesis that height can be judged from faces, but not that it can be judged more accurately from 3D images than 2D images (see also Figure 2).

### Model 3: BMI

Full results for Model 3 are shown in Table 2. The mean weight rating was 3.99. There was a significant positive effect of stimulus gender (estimate = 0.04, 95% CI = [0.01, 0.07], *p* = .011), indicating that male stimulus faces received higher weight ratings than female stimulus faces. There was also a significant positive effect of actual BMI (estimate = 0.44, 95% CI=[0.37, 0.51], *p* < .001); a difference in BMI equivalent to the sample range led to an increase in perceived weight by 2.64 points on the 7-point scale. The model also showed a negative effect of stimulus type (estimate = −0.02, 95% CI=[−0.03, −0.01], *p* = .003), indicating that 3D faces received slightly lower weight ratings than 2D faces. The interaction of actual BMI and stimulus type was not significant (estimate = 0.02, 95% CI=[−0.03, 0.07], *p* = .381). These results provide evidence for our hypothesis that BMI can be judged from faces, but not that it can be judged more accurately from 3D images than 2D images (see also Figure 2).

As we had to deviate from our pre-registered protocol by moving the study online and splitting our stimulus set into three subsets, not all stimuli were rated by all participants as originally planned. To account for potential individual differences in scale use, we repeated our main analyses including random effects for individuals. These analyses showed the same pattern of results as found in the pre-registered aggregated analyses. Full model specifications and results can be found in the supplemental material (https://osf.io/wz5nc/).

### Robustness Checks

To check that potential ‘carry over’ effects on ratings that could be introduced by having participants rate both 2D and 3D versions of the same faces do not affect results, we had planned to repeat our three main analyses using ratings only from the first block of trials for each rater.

Results can be found in the supplemental material (https://osf.io/wz5nc/). The most notable difference to results from our main analyses was that for height, the interaction of stimulus type and trait value was significant here, but in the opposite direction to what we had predicted: height appeared to be more accurately perceived from 2D compared to 3D images (estimate = −0.07, 95% CI =[−0.13, −0.01], *t* = −2.18 *p* = .031). It is worth noting though that blocks were not perfectly balanced; the order in which blocks were presented was randomized through the online interface. This means that for data from this first block, number of ratings per stimulus face ranged from as little as 6 up to 16.

## Discussion

Ratings of height and weight from faces were positively and significantly correlated with measured (i.e., actual) height and BMI, respectively. This is consistent with previous research suggesting that people can judge height and weight (Coetzee et al., 2009, 2010; Holzleitner et al., 2014; Re et al., 2012, 2013; Tinlin et al., 2013) somewhat accurately from faces. By contrast, strength ratings were not significantly positively correlated with measured strength, suggesting that people were relatively poor at assessing strength from faces. This null result is somewhat surprising, given some previous research has suggested the existence of facial cues of upper body strength (Sell et al., 2008). However, the null result that we saw for strength is consistent with previous work suggesting other facial cues, such as those related to body size, can cause misperceptions of strength from facial appearance (Holzleitner & Perrett, 2016).^3^

None of our analyses revealed any evidence that physical characteristics of the individual photographed could be judged more accurately from 3D than 2D images. In other words, none of our analyses showed significant moderating effects of stimulus type (3D versus 2D) on the strength of the relationships between measured and rated physical traits. Although it is perhaps intuitively surprising that physical characteristics related to body size and strength are not captured better by 3D than 2D images, our results are consistent with previous research that found perceptual ratings of 3D and 2D faces were very highly correlated (Tigue et al., 2012; Trebicky et al., 2018). Further work is needed to establish whether the patterns of results observed in the current study hold for stimuli in which non-face cues, such as neck size, are visible in the stimuli. Similarly, further work is needed to establish whether our results for BMI hold when body weight is the target variable, rather than BMI.

In summary, our analyses present further evidence that height and weight can be judged somewhat accurately from faces, but provide little compelling evidence for the claim that cues of upper body strength are visible in faces. Perhaps more importantly, our results suggest that there may be little to be gained by using 3D face scans to investigate perceptions of the facial correlates of physical characteristics.

## Footnotes

1 Our pre-registered sample size was n = 189 (125 women, and 64 men). We lost face images of two participants due to poor image quality, and another five participants due to lack of consent required to conduct the study online, see below.

2 We had initially planned to create GIFs with a frame rate of 12 frames per second. These GIFs were about 7MB and had a duration of 7.5s. Moving the study online meant file size was a concern due to remote loading times, and length of videos due to expected attrition. To reduce file size, we moved from GIF file format to M4V and we increased frame rate from 12 to 18. This reduced file size to about 1.5MB and playtime to about 5s per stimulus face.

3 Holzleitner and Perrett (2016) suggested that perception of strength is driven by an overgeneralization of facial cues to bulk. In line with this reasoning, we found that in the current sample perception of strength was predicted by BMI (estimate=0.17, 95% CI=[0.11, 0.23], *t*=5.39, *p*<.001, see supplemental analyses at https://osf.io/wz5nc/).
